# Age-dependent roles of peroxisomes in the hippocampus of a transgenic mouse model of Alzheimer’s disease

**DOI:** 10.1186/1750-1326-8-8

**Published:** 2013-02-02

**Authors:** Francesca Fanelli, Sara Sepe, Marcello D’Amelio, Cinzia Bernardi, Loredana Cristiano, AnnaMaria Cimini, Francesco Cecconi, Maria Paola Ceru', Sandra Moreno

**Affiliations:** 1 Department of Biology-LIME, University “Roma Tre”, viale Marconi 446, 00146, Rome, Italy; 2 IRCCS S. Lucia Foundation, via del Fosso di Fiorano 65, 00143, Rome, Italy; 3 University Campus Bio-Medico, via Alvaro del Portillo 21, 00128, Rome, Italy; 4 Department of Radiological Sciences and Laboratory Medicine, UOC Pathological Anatomy, San Filippo Neri Hospital, via Martinotti 20, 00135, Rome, Italy; 5 Department of Life, Health and Environmental Sciences, University of L’Aquila, piazzale Salvatore Tommasi 1, 67100, Coppito, (AQ), Italy; 6 Department of Biology, University of Rome ‘Tor Vergata’, via della Ricerca Scientifica, 00133, Rome, Italy

**Keywords:** Peroxisome, Brain aging, Alzheimer’s disease, Neurodegeneration, Oxidative stress, Lipid metabolism, Catalase, Superoxide dismutase, Glutathione peroxidase, Acyl-CoA beta-oxidation

## Abstract

**Background:**

Alzheimer’s Disease (AD) is a progressive neurodegenerative disease, especially affecting the hippocampus. Impairment of cognitive and memory functions is associated with amyloid β-peptide-induced oxidative stress and alterations in lipid metabolism. In this scenario, the dual role of peroxisomes in producing and removing ROS, and their function in fatty acids β-oxidation, may be critical. This work aims to investigating the possible involvement of peroxisomes in AD onset and progression, as studied in a transgenic mouse model, harboring the human Swedish familial AD mutation. We therefore characterized the peroxisomal population in the hippocampus, focusing on early, advanced, and late stages of the disease (3, 6, 9, 12, 18 months of age). Several peroxisome-related markers in transgenic and wild-type hippocampal formation were comparatively studied, by a combined molecular/immunohistochemical/ultrastructural approach.

**Results:**

Our results demonstrate early and significant peroxisomal modifications in AD mice, compared to wild-type. Indeed, the peroxisomal membrane protein of 70 kDa and acyl-CoA oxidase 1 are induced at 3 months, possibly reflecting the need for efficient fatty acid β-oxidation, as a compensatory response to mitochondrial dysfunction. The concomitant presence of oxidative damage markers and the altered expression of antioxidant enzymes argue for early oxidative stress in AD. During physiological and pathological brain aging, important changes in the expression of peroxisome-related proteins, also correlating with ongoing gliosis, occur in the hippocampus. These age- and genotype-based alterations, strongly dependent on the specific marker considered, indicate metabolic and/or numerical remodeling of peroxisomal population.

**Conclusions:**

Overall, our data support functional and biogenetic relationships linking peroxisomes to mitochondria and suggest peroxisomal proteins as biomarkers/therapeutic targets in pre-symptomatic AD.

## Background

Alzheimer’s disease (AD) is the most common form of dementia, characterized by progressive neurodegeneration, particularly affecting the hippocampal formation. Impairment of cognitive and memory functions is associated with amyloid β-peptide (Aβ) accumulation, increased oxidative stress, lipid metabolism alteration, and inflammation [1-5].

The central role of peroxisomes in reactive oxygen species (ROS) metabolism has emerged since their discovery [6]. In fact, peroxisomes participate in both ROS generation and removal, under physiological or pathological conditions. Moreover, peroxisomes are involved in a wide range of catabolic and anabolic functions, including β-oxidation of very long chain fatty acids (VLCFAs) [7,8], biosynthesis of polyunsaturated fatty acids and plasmalogens [9,10], and calcium homeostasis [11,12]. Emergent studies have revealed that peroxisomes can also function as intracellular signaling compartments and organizing platforms that orchestrate important developmental decisions from inside the cell [13]. These dynamic and versatile organelles respond to physiological and pathological changes in cellular environment by adapting their morphology, number and enzyme content accordingly [14]. Peroxisomal dysfunction has been shown to be associated with cellular aging as well as with age-related degenerative diseases [13]. The potential role of peroxisomes in human AD, especially in relationship with lipid metabolism, has recently been suggested [15]. Indeed, a decrease in plasmalogens and, conversely, an increase in VLCFAs, were described in cases with advanced Braak stages [16]. Moreover, in an *in vitro* model of advanced AD a decrease of peroxisomes in hippocampal neurons was reported, while induction of peroxisomal proliferation attenuated Aβ-dependent toxicity [17]. We previously demonstrated that peroxisomes are involved in early stages of AD, as studied either *in vivo*, in a transgenic mouse model [18], or *in vitro*, on Aβ treated cortical neurons [19].

The aim of the present work was to investigate the role of peroxisomes during the progression of AD and in normal aging. To this purpose, we utilized the Tg2576 (Tg) mouse model, compared to its wild-type (WT) counterpart. Differently from other mouse models, this strain displays a slowly progressive AD pathology, offering the opportunity to study even subtle age-dependent alterations [20-23]. By combined molecular and morphological approaches, we examined the expression of peroxisome-related proteins in the hippocampal formation at early and advanced AD stages (3, 6, 9, 12, and 18 months). We focused on the CA1 hippocampal region, as pyramidal cells in this field are the most profoundly affected cell type in human AD [24]. Specifically, the expression and immunolocalization patterns of peroxisomal membrane protein of 70 kDa (PMP70), peroxin 14p (Pex14p), catalase (CAT), acyl-CoA oxidase 1 (AOX), and 3-ketoacyl-CoA thiolase (THL) were studied, to get an insight into the biogenesis and functioning of peroxisomes in the diseased brain. Given the involvement of peroxisomes in the maintenance of the redox status, antioxidant enzymes other than CAT, namely selenium-dependent glutathione peroxidase (GPX1), Cu,Zn-superoxide dismutase (SOD1), and Mn-superoxide dismutase (SOD2) were also investigated in Tg animals and in their WT littermates. It is worth recalling that these proteins, though mainly localized to different cell compartments, were also found in peroxisomes [25]. At selected stages, markers for oxidative damage to lipids and nucleic acids - acrolein and 8-hydroxy(deoxy)guanosine (8-OH(d)G), respectively - were also studied.

Notably, the size and functions of peroxisomal population are regulated by a class of ligand-activated transcription factors (peroxisome proliferator-activated receptors, PPARs) [26]. Among these, we studied PPARα which is directly involved in peroxisomal induction [27], plays a neuroprotective role in age-related inflammation [28] and enhances memory consolidation [29]. It is worth-mentioning in this context that PPARα specific agonists have been demonstrated to exert a neuroprotective action against Aβ-mediated toxicity *in vitro*[17]. Finally, we examined the expression of PPARγ coactivator-1γ (PGC-1α), in view of its synergism with PPARα and its implication in peroxisomal remodeling and biogenesis [30,31].

## Results

The distribution of peroxisomes in Tg and WT hippocampal formation from 3-, 6-, 9-, 12-, and 18-month-old mice was investigated by analyzing the expression of peroxisomal membrane (PMP70, Pex14p) and matrix (CAT, AOX, THL) proteins. Markers of oxidative damage to nucleic acids (8-OH(d)G) and to lipids (acrolein), as well as antioxidant enzymes (SOD1, SOD2, GPX1), were also studied. Further, to get an insight into the transcriptional regulation of these genes, PPARα and PGC-1α expression was examined. At selected stages, the presence of PMP70 and PPARα in neuronal and/or astroglial cells was studied by immunofluorescence and their ultrastructural localization was investigated by immunoelectron microscopy.

### Peroxisomal membrane proteins: Pex14p and PMP70

**Figure 1:**
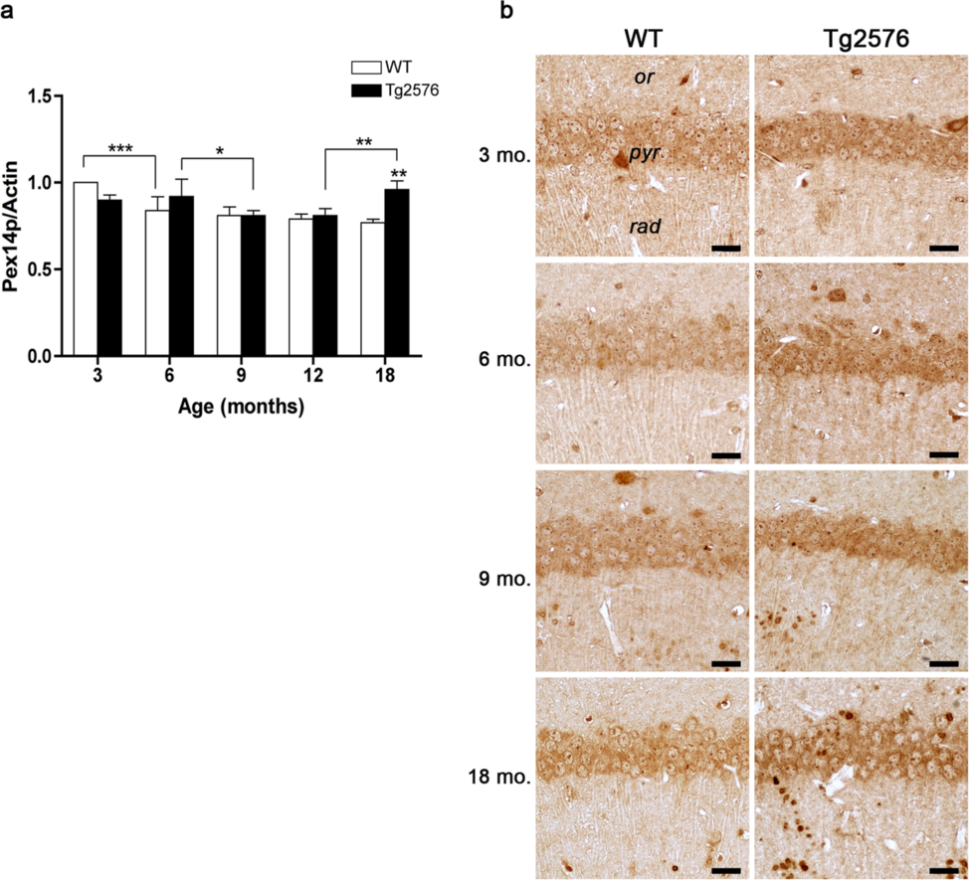
**Pex14p protein levels and distribution in the hippocampus of WT and Tg mice.** (**a**) Densitometric values of Pex14p WB, performed on hippocampal protein extracts of 3-, 6-, 9-, 12-, and 18-month-old WT and Tg mice. Data are expressed as mean ± SD. *P <0.05; **P <0.01; ***P <0.001. (**b**) Pex14p immunohistochemical localization in CA1 hippocampal field of 3-, 6-, 9-, and 18-month-old WT and Tg mice*. or, stratum oriens; pyr, stratum pyramidale; rad, stratum radiatum.* Scale bars, 25 μm.

Pex14p is a membrane-anchored peroxin, involved in the peroxisomal import of matrix proteins [32-34], and thus considered a dependable marker for peroxisomal number [35]. Pex14p hippocampal levels, as assessed by densitometric analysis of WB (Figure 1a), show no genotype-dependent differences throughout the examined period, except for 18 months of age, when remarkably higher protein concentration in Tg, compared to WT, is detected. Rather, age-related decreases are observed in both normal and diseased animals, namely between 3 and 6 months in the former, and between 6 and 9 months in the latter. This pattern is consistent with Pex14p immunohistochemical staining of pyramidal neurons in CA1 hippocampal field (Figure 1b).

**Figure 2:**
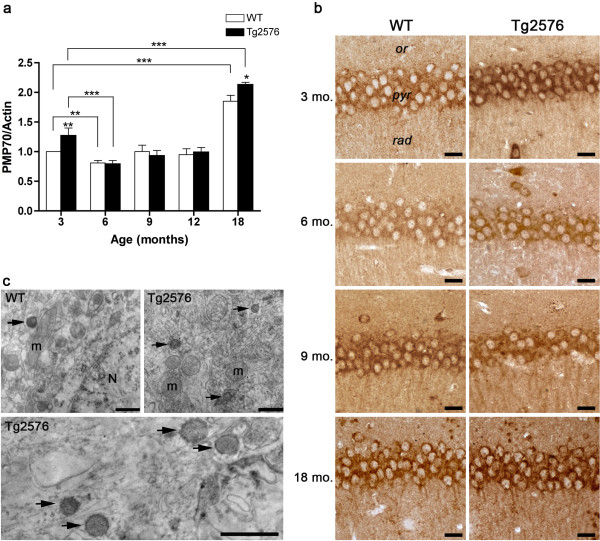
**PMP70 protein levels and distribution in the hippocampus of WT and Tg mice.** (**a**) Densitometric values of PMP70 WB, obtained analyzing the hippocampal protein extracts of 3-, 6-, 9-, 12-, and 18-month-old WT and Tg mice. Data are expressed as mean ± SD. *P <0.05; **P <0.01; ***P <0.001. (**b**) Immunohistochemical distribution of PMP70 in CA1 hippocampal field of 3-, 6-, 9-, and 18-month-old WT and Tg mice. *or, stratum oriens; pyr, stratum pyramidale; rad, stratum radiatum.* Scale bars, 25 μm. (**c**) PMP70 pre-embedding immunoelectron microscopy of 3-month-old CA1 pyramidal neurons of WT and Tg animals. In both genotypes positive peroxisomes (arrows) are observed and they appear more numerous in the cytoplasm of Tg neurons. N, neuronal nucleus; m, mitochondrion. Scale bars, 1 μm.

PMP70, a major component of mammalian peroxisomal membranes, is also considered as a good marker for the overall size of peroxisomal population [36]. This ATP-binding cassette transporter, also known as ABCD3, is suggested to be responsible for the metabolic transport of long and branched-chain fatty acyl-CoAs [37]. Our WB data (Figure 2a) show a significant PMP70 induction in 3-month-old Tg hippocampus compared to control. A decrease at 6 months of age ensues in both genotypes, being especially dramatic in the diseased mice. Neither age- nor genotype-related variations are detected at 9 and 12 months, while a peak of PMP70 protein levels is observed at 18 months, particularly in the pathological genotype.

PMP70 immunohistochemical results on the CA1 hippocampal field are in agreement with molecular data (Figure 2b). Indeed, 3-month-old Tg pyramidal cell layer displays stronger immunoreactivity than its WT counterpart, while, at 6 months, decreased immunostaining is observed in both genotypes. At 9–12 months of age, immunostaining levels remain stable, and they remarkably increase in 18-month-old hippocampus, especially in the somata of Tg pyramidal neurons. PMP70 pre-embedding immunoelectron microscopy allowed us to identify positive peroxisomes in 3-month-old CA1 pyramidal neurons, in both WT and Tg animals (Figure 2c). The immunoreaction product appears confined to the membrane, as expected. Consistent with molecular and immunohistochemical data, ultrastructural analysis shows numerous PMP70 immunoreactive peroxisomes in hippocampal cells of young Tg mice.

**Figure 3:**
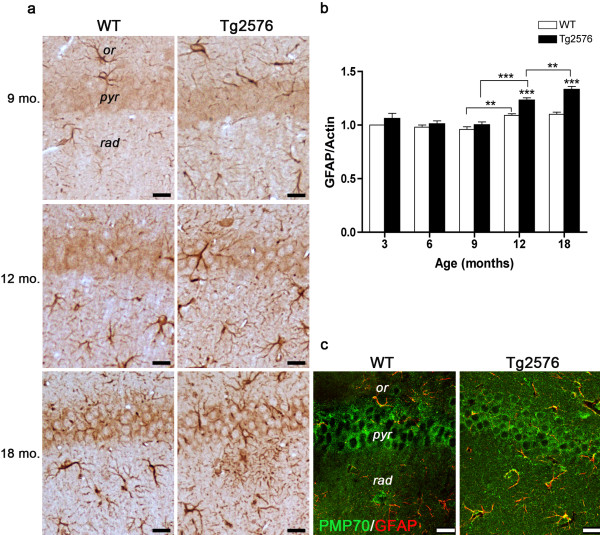
**GFAP protein levels and distribution in the hippocampus of WT and Tg mice.** (**a**) GFAP immunohistochemical localization in CA1 hippocampal field of 9-, 12-, and 18-month-old WT and Tg mice*. or, stratum oriens; pyr, stratum pyramidale; rad, stratum radiatum.* Scale bars, 25 μm. (**b**) Densitometric analysis of GFAP WB performed on hippocampal protein extracts of 3-, 6-, 9-, 12-, and 18-month-old mice. Values are expressed as mean ± SD. **P < 0.01; ***P < 0.001. (**c**) Double immunofluorescence of PMP70 (green) in combination with GFAP (red) in the CA1 hippocampal field of 18-month-old WT and Tg brain. Several PMP70^+^/GFAP^+^ cells (yellow) are especially numerous in the pathological genotype. Scale bars, 25 μm.

The strong PMP70 immunoreactivity observed at 18 months suggests possible contribution by astrogliosis to peroxisome numerical increase. To address this issue, we performed GFAP immunohistochemistry and immunoblotting (Figures 3a and b), as well as double immunofluorescence of PMP70 in combination with GFAP (Figure 3c). As expected, astrogliosis is present in the aging hippocampus, particularly in the Tg animals, and also associates with senile plaques. Confocal images of 18-month-old WT and Tg CA1 fields show bright PMP70 immunofluorescence in GFAP^–^ and GFAP^+^ cells, in both genotypes. PMP70^+^/GFAP^+^ cells are especially numerous in Tg, demonstrating that peroxisomal increase in the AD senescent hippocampus is importantly contributed by astroglial cells.

### Peroxisomal fatty acid β-oxidation enzymes

**Figure 4:**
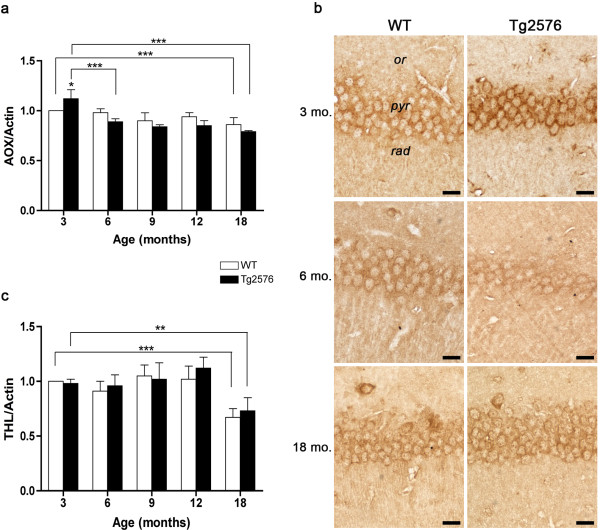
**Peroxisomal fatty acid β-oxidation enzymes in the hippocampus of WT and Tg mice.** (**a**, **c**) Densitometric values of AOX and THL WB performed on hippocampal protein extracts of 3-, 6-, 9-, 12-, and 18-month-old WT and Tg brains. Data are expressed as mean ± SD. *P <0.05; **P <0.01; ***P <0.001. (**b**) AOX immunolocalization in CA1 hippocampal field of 3-, 6-, and 18-month-old WT and Tg brains. *or, stratum oriens; pyr, stratum pyramidale; rad, stratum radiatum.* Scale bars, 25 μm.

Even though the important role of peroxisomal β-oxidation in brain development and functioning is well recognized [8], no information on this metabolic pathway in AD is presently available. We therefore analyzed the expression of two major peroxisomal β-oxidation enzymes, namely AOX and THL at the onset and during the progression of AD. The former is the rate-limiting enzyme catalyzing the first step of the cycle, while THL is the last enzyme of the pathway. In 3-month-old Tg hippocampus, AOX expression is significantly higher than in its WT counterpart (Figure 4a). Starting from 6 months, protein levels significantly decrease and remain relatively low throughout the progression of disease. This pattern is supported by AOX immunohistochemical analysis, showing intense immunoreactivity in CA1 pyramidal cell somata of 3-month-old Tg hippocampus, not observed at subsequent stages (Figure 4b). The expression pattern of THL displays no genotype- or age-linked variations, except for 18 months, when hippocampal levels are strongly decreased in both genotypes, suggesting age-dependent impairment of peroxisomal β-oxidation (Figure 4c).

### ROS-scavenging enzymes: CAT, GPX1, SOD1 and SOD2

**Figure 5:**
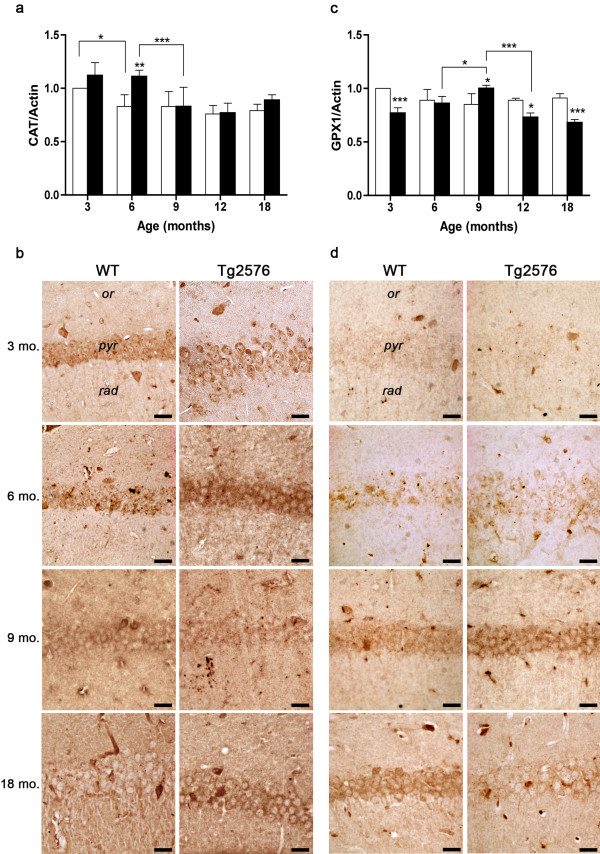
**H**_**2**_**O**_**2**_**-scavenging enzymes in WT and Tg hippocampus.** (**a**, **c**) Densitometric values of CAT and GPX1 WB performed on hippocampal protein extracts of 3-, 6-, 9-, 12-, and 18-month-old WT and Tg brains. Values are expressed as mean ± SD. *P <0.05; **P <0.01; ***P <0.001. (**b**, **d**) CAT and GPX1 immunolocalization in CA1 hippocampal field of 3-, 6-, 9- and 18-month-old WT and Tg brains. *or, stratum oriens; pyr, stratum pyramidale; rad, stratum radiatum.* Scale bars, 25 μm.

Peroxisomes play a pivotal role in the protection against ROS, mainly through their CAT activity, but also thanks to their content in SOD1, SOD2, and GPX1 [25,38,39]. High CAT levels are found in 3-month-old hippocampus irrespective of the genotype, while at 6 months significantly higher values are observed in Tg, as compared to WT (Figure 5a). At 9 months, CAT expression in the Tg decreases to WT levels, which are maintained at later ages in both genotypes. These data are confirmed by immunohistochemical results, CA1 pyramidal neurons being especially CAT positive in 6-month-old Tg hippocampus (Figure 5b).

The other major H_2_O_2_–scavenging enzyme, GPX1, shows no significant variations during normal aging, while differences in its expression levels are seen in the pathological genotype, during AD progression. Indeed, at 3 months, GPX1 expression in Tg is lower than in WT, increasing thereafter, to reach significantly higher levels than WT at 9 months. In the aging Tg hippocampus a progressive decrease in GPX1 protein, below WT levels, is observed (Figure 5c). Immunohistochemical data are consistent with WB data (Figure 5d).

**Figure 6:**
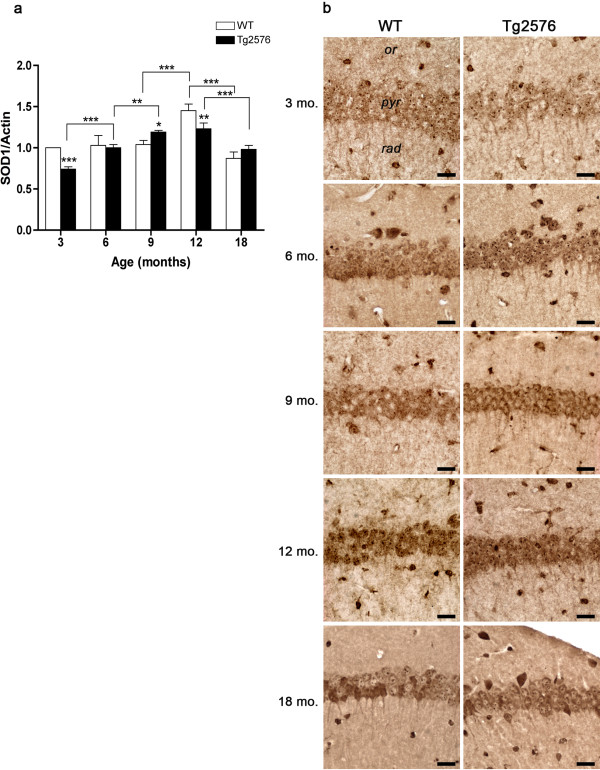
**SOD1 protein levels and distribution in WT and Tg hippocampus.** (**a**) Densitometric values of SOD1 WB performed on hippocampal protein extracts of 3-, 6-, 9-, 12- and 18-month-old WT and Tg brains. Values are expressed as mean ± SD. *P <0.05; **P <0.01; ***P <0.001. (**b**) SOD1 immunolocalization in CA1 hippocampal field of 3-, 6-, 9-, 12- and 18-month-old WT and Tg brains. *or, stratum oriens; pyr, stratum pyramidale; rad, stratum radiatum.* Scale bars, 25 μm.

**Figure 7:**
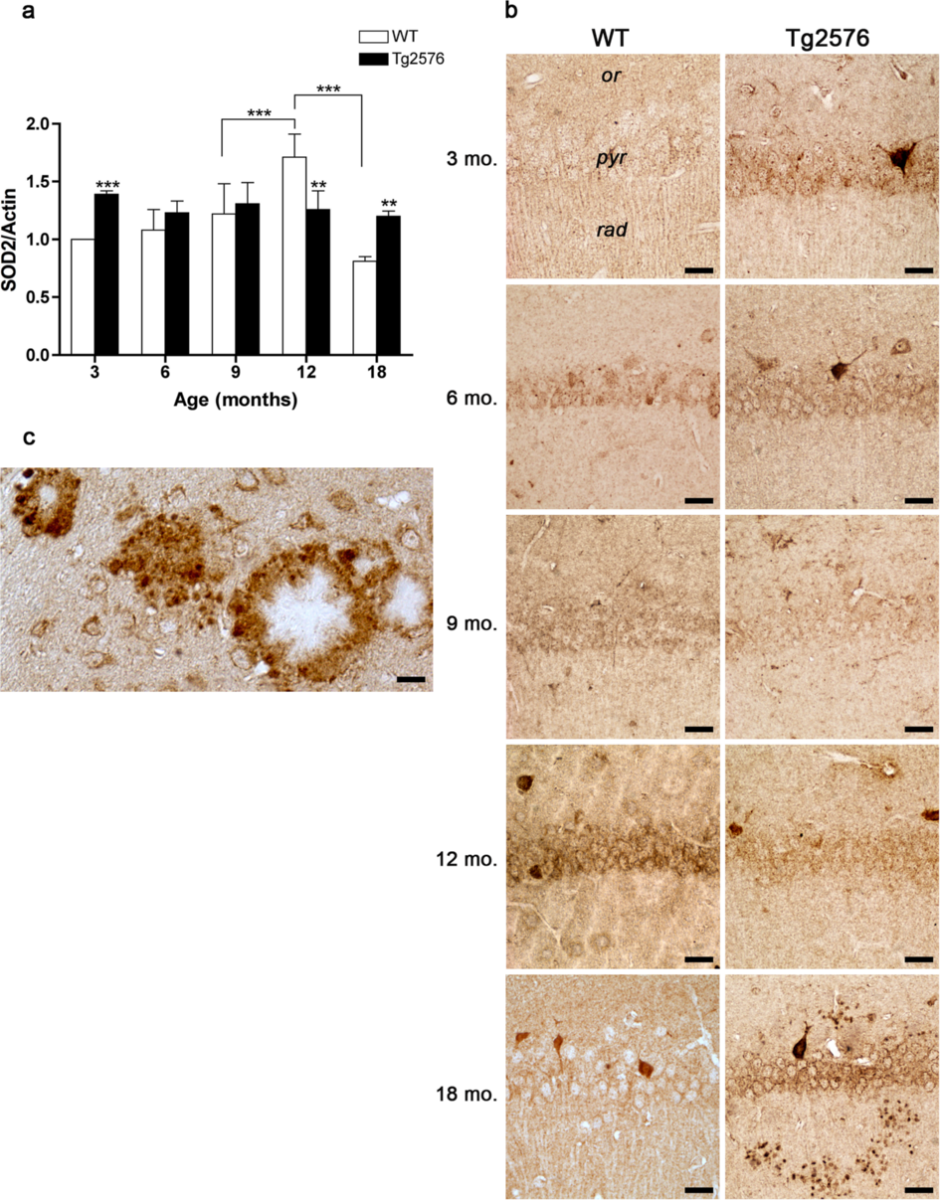
**SOD2 protein levels and distribution in WT and Tg hippocampus.** (**a**) Densitometric values of SOD2 WB performed on hippocampal protein extracts of 3-, 6-, 9-, 12- and 18-month-old WT and Tg brains. Values are expressed as mean ± SD. **P <0.01; ***P <0.001. (**b**) SOD2 immunolocalization in CA1 hippocampal field of 3-, 6-, 9-, 12- and 18-month-old WT and Tg brains. *or, stratum oriens; pyr, stratum pyramidale; rad, stratum radiatum.* Scale bars, 25 μm. (**c**) Higher magnification showing SOD2 immunopositive glial cells around senile plaques in 18-month-old Tg hippocampus. Scale bar, 15 μm.

The molecular and morphological expression patterns of the two enzymes responsible for the dismutation of the superoxide anion to hydrogen peroxide – SOD1 and SOD2 – are shown in Figures 6 and 7, respectively. As to SOD1 (Figure 6a, b), in 3-month-old Tg hippocampus its levels are significantly lower than in WT, but progressively increase thereafter, reaching at 9 months significantly higher levels than its WT counterpart. SOD1 expression remains stable in the Tg at 12 months of age, when WT levels are instead maximally increased, and sharply decreases at 18 months in both genotypes.

SOD2 expression pattern during normal aging (Figure 7a, b) closely resembles that of SOD1 in WT hippocampus, while in Tg mice no age-dependent variations are observed. Interestingly, SOD2 levels are significantly higher in 3- and in 18-month-old Tg hippocampus, compared to their WT counterparts. Remarkably, at 18 months, SOD2 mainly localizes to glial cells surrounding senile plaques in Tg hippocampus (Figure 7c).

### Oxidative stress markers: acrolein and 8-OH(d)G

**Figure 8:**
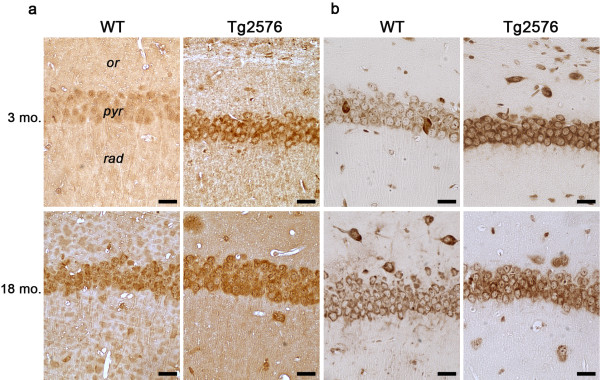
**Immunolocalization of oxidative stress markers at early and advanced AD stages.** (**a**, **b**) Acrolein and 8-OH(d)G in CA1 hippocampal field of 3- and 18-month-old WT and Tg mice. *or, stratum oriens; pyr, stratum pyramidale; rad, stratum radiatum.* Scale bars, 25 μm.

To evaluate possible oxidative stress occurring in the hippocampus during normal and pathological aging, markers of oxidative damage to biomolecules were investigated by immunohistochemistry. Antibodies to acrolein, marker of lipid peroxidation (Figure 8a), and to 8-OH(d)G, marker of DNA/RNA oxidative modifications (Figure 8b), were tested at 3 and 18 months, as representative of early and advanced AD stages.

We found a similar immunoreactivity pattern for both markers, strongly related to the genotype, in that intense labeling in Tg CA1 pyramidal layer was observed, as early as 3 months, when WT neurons are faintly immunopositive in their cytoplasm. In 18-month-old hippocampus, oxidative damage markers are detected in both WT and Tg CA1 pyramidal cells, even though the former are consistently less immunoreactive than the latter. Intriguingly, the intensity of 8-OH(d)G immunoreaction at 18 months in Tg CA1 neurons appears lower than at 3 months, suggesting a lower degree of oxidative damage to nucleic acids in late AD.

### PPARα and PGC-1α

**Figure 9:**
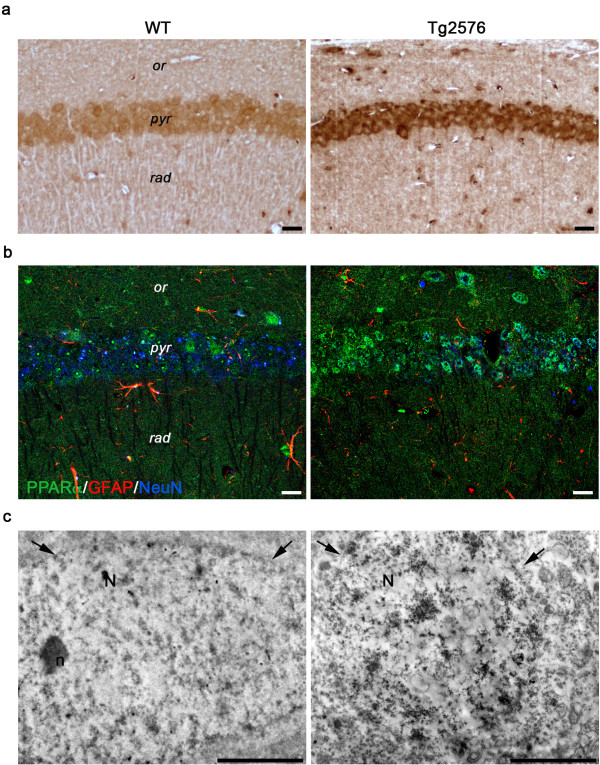
**PPARα immunolocalization in 3-month-old WT and Tg mice.** (**a**) PPARα immunohistochemistry in CA1 pyramidal cell layer shows a higher immunoreactivity in Tg hippocampus, than in its WT counterpart. *or, stratum oriens; pyr, stratum pyramidale; rad, stratum radiatum.* Scale bars, 25 μm. (**b**) Confocal image of triple immunofluorescence for PPARα (green) in combination with GFAP (red) and NeuN (blue) demonstrates the presence of PPARα-positive neurons and astrocytes. Note the brightness of green signal in the Tg section. Scale bars, 25 μm. (**c**) PPARα pre-embedding immunoelectron microscopy of CA1 pyramidal neurons shows the nuclear localization of the immunoreaction product, especially concentrated in the Tg nucleus. Arrows indicate the nuclear envelope. N, nucleus; n, nucleolus. Scale bars, 2 μm.

Since the expression and activity of PPARα, major regulator of peroxisome biogenesis, is influenced by oxidative stress [28], we also addressed the putative involvement of this transcription factor at the onset and at late stages of AD. PPARα immunostaining of CA1 pyramidal cell layer shows significantly higher levels in 3-month-old Tg hippocampus, compared to its WT counterpart (Figure 9a). Triple immunofluorescence experiments of PPARα in combination with the neuronal marker NeuN and the astroglial marker GFAP, confirm high expression of the receptor in 3-month-old Tg neuronal cell nuclei and demonstrate its presence also in glial cell nuclei (Figure 9b). Pre-embedding immunoelectron microscopy further confirms the massive presence of PPARα within the nucleus of Tg hippocampal pyramidal neurons (Figure 9c).

**Figure 10:**
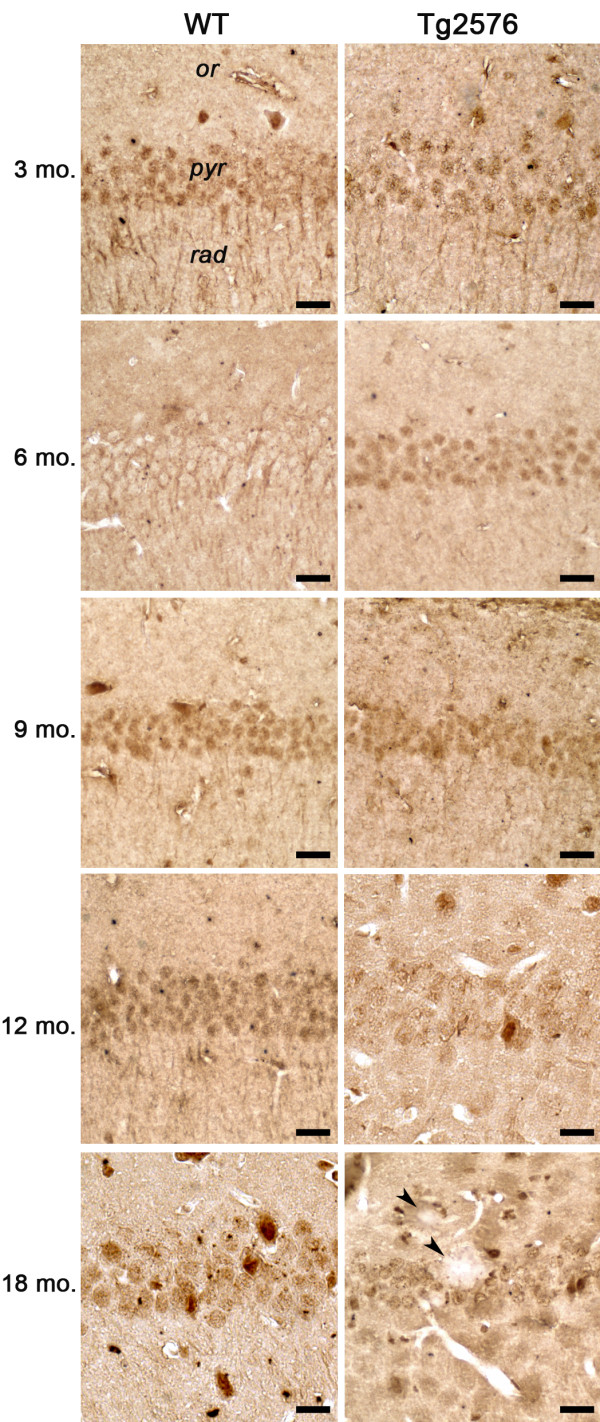
**PGC-1α immunohistochemistry in CA1 hippocampal field of 3, 6-, 9-, 12- and 18-month-old WT and Tg mice.** Arrowheads, Aβ plaques. *or, stratum oriens; pyr, stratum pyramidale; rad, stratum radiatum.* Scale bars, 25 μm.

Given the growing evidence for a PGC-1α-mediated transcriptional regulation in response to oxidative stress and in view of its roles in peroxisome biogenesis [31] and in promoting the nonamyloidogenic pathway of APP [40], we analyzed the immunohistochemical localization of this coactivator in the hippocampal CA1 region (Figure 10). At 3 months of age, PGC-1α appears localized in both the nucleus and the cytoplasm, including dendrites extending in the stratum radiatum, of WT and Tg hippocampus. Interestingly, at 6 months, immunoreactivity is highly concentrated in Tg neuronal nuclei, while in WT hippocampus nuclear localization is observed at 9 months. At the latest age considered, a general decrease in immunoreactivity is detected.

## Discussion

The aim of the present study was to investigate the role of peroxisomes in AD onset and progression. In a previous work [18], peroxisomal involvement in early AD was demonstrated in the Tg2576 mouse model, also used in this work. Differently from that study, in which male animals were analyzed, we here utilized females, since epidemiological and histopathological data indicate this as the most AD-affected gender in mice and humans [41,42]. We focused on the hippocampal formation, i.e., the primarily affected area of the diseased brain [43]. The expression of peroxisome-related proteins was studied by combined molecular and morphological approaches, at the onset (3 months) and during AD progression (6, 9, 12, 18 months) in Tg animals, compared to their WT counterparts.

Our results demonstrate that significant peroxisomal alterations occur in Tg mice, as early as 3 months. These early changes are consistent with behavioral, electrophysiological, ultrastructural and molecular modifications reported in this model at the same age [22]. Indeed, PMP70, long been considered marker of the numerical size of peroxisomal population [36] is upregulated, as assessed by WB. Consistently, PMP70 immunohistochemistry reveals especially intense immunostaining in the pyramidal layer of Tg hippocampal CA1 subdivision, and immunoelectron microscopy shows numerous positive peroxisomes in the somata of these neurons.

Differently from PMP70, the biogenesis marker Pex14p, also used to evaluate peroxisome number [35], is unchanged in 3-month-old Tg animals. The absence of correlation between PMP70 and Pex14p levels in early AD could either indicate specific concentration of PMP70 in a numerically unchanged peroxisomal population, or impoverishment of Pex14p in proliferated peroxisomes. In either case, the altered content in these proteins likely results in profound modifications of peroxisomal functions. Interestingly, Pex14p has recently been involved in microtubule-based peroxisome motility [44], suggesting that at this early AD stage, peroxisomes, endowed with a relatively low Pex14p/PMP70 ratio are altered in their transport along neurites. To this respect, it is worth mentioning that a recent work from Berger’s group demonstrates an increased peroxisomal volume density in neuronal cell bodies of human AD brain, associated with impaired peroxisome trafficking [16].

The specific increase in early AD of PMP70, presumably involved in fatty acyl-CoA import across peroxisomal membranes [37], could reflect the need for a more efficient acyl-CoA β-oxidation. In agreement with this hypothesis, AOX, rate-limiting enzyme of peroxisomal β-oxidation pathway, is upregulated in 3-month-old Tg hippocampus. This increase could thus represent a compensatory response to early Aβ-mediated mitochondrial insult, to cope with the impaired energy metabolism, occurring in AD [45,46]. Indeed, abnormalities in glucose metabolism/mitochondrial function are invariant features of early AD [46-49]. Consistently, positron emission tomography studies in AD patients report abnormal cerebral glucose utilization, particularly in the hippocampus, decades prior to the onset of histopathological and clinical features [50]. A consequent shift in brain metabolism, from a primarily aerobic glycolysis pathway to a ketogenic/fatty acid β-oxidation pathway is thought to occur [51]. In this context, we suggest that peroxisomal β-oxidation contributes to such metabolic change, providing mitochondria with acetyl-CoA and fatty acyl substrates. Induction of peroxisomal β-oxidation pathway may also be neuroprotective, by enhancing the clearance of potentially toxic VLCFAs and by promoting the biosynthesis of the neuroprotective docosahexaenoic acid (DHA) [15]. Relevantly, elevated levels of VLCFA have been found in human AD brain lesions [16] and the ability of these fatty acids to trigger oxidative stress and mitochondrial dysfunction on neural cells has recently been demonstrated [52,53].

A biogenetic/metabolic relationship between peroxisomes and mitochondria has long been established and recently emphasized [13,30,31]. In yeast, experimentally induced or age-associated mitochondrial dysfunction activates the so-called retrograde (RTG) signaling pathway, leading to the transcription of RTG-target genes, including those required for peroxisome biogenesis and function [13]. Our results showing increased PPARα nuclear immunoreactivity allow us to speculate that this gene be included among the above mentioned RTG-target genes. The increased PPARα expression could in turn be responsible for the induction of its target genes PMP70 and AOX [54,55]. Even though the molecular triggers of PPARα activation remain to be determined in our model, endogenous production of PPARα ligands, *e.g.*, oxidized lipid molecules, is likely to occur due to Aβ-mediated ROS generation.

While, in principle, AOX induction in 3-month-old Tg hippocampus may represent a positive response to enhanced need for lipid substrates to be processed in mitochondria, this induction may also result in increased H_2_O_2_ production by the oxidase. Moreover, mitochondrial respiration supported by fatty acids generates substantial rates of ROS production [56], thus contributing to cellular redox status alteration. In this context, even the observed early increase of SOD2 expression, in agreement with reports demonstrating induction of mitochondrial genes in young Tg mice [57], may contribute itself to redox imbalance, through enhanced conversion of superoxide anion to H_2_O_2_.

Notably, GPX1, the major cytosolic H_2_O_2_–scavenging enzyme, is significantly down-regulated in our early AD samples, further exacerbating imbalance between generation and removal of hydrogen peroxide. GPX1 seems especially critical in AD, since its deletion increases susceptibility of neurons to Aβ-mediated damage, while its overexpression protects against neurodegeneration [58,59]. The postulated pro-oxidant environment likely results in post-translational modifications of GPX1 itself, leading to its irreversible inactivation [60]. Even the low levels of SOD1 detected in the young Tg hippocampus may be explained taking into account that this cytosolic protein is selectively damaged by H_2_O_2_[61]. Importantly, SOD1 deficiency in Tg2576 mice has been associated with oxidative damage, accelerated Aβ oligomerization and memory impairment [62].

Therefore, our data strongly support the idea that oxidative stress is the primary culprit in AD pathogenesis [63]. Consistently, 8-OH(d)G and acrolein, markers of oxidative modification to biomolecules [64,65], show DNA/RNA oxidation and lipoperoxidation in the hippocampus of young Tg mice. Interestingly, 8-OH(d)G immunostaining is predominantly localized in the cytoplasm, suggesting that mitochondrial nucleic acids are primarily affected. To our knowledge, this is the first report of such a modification occurring at 3 months in this mouse strain. In this context, it is worth mentioning that acrolein has been suggested not only as a marker of lipoperoxidation, but also as initiator of oxidative stress [65].

Between 3 and 6 months of age, most peroxisomal markers decrease in WT hippocampal formation, indicating a numerical reduction and/or a metabolic change of the organelles in the physiological course of maturity. By contrast, in Tg mice of the same age, when several hallmarks of AD pathology make their appearance [21], marker-specific patterns are observed. In particular, while high levels of CAT and Pex14p are maintained, PMP70 and AOX are significantly decreased, suggesting metabolic, rather than numerical, modifications of peroxisomes. These changes, indicating decreased efficiency of peroxisomal β-oxidation, presumably lead to VLCFA accumulation, consistent with what reported in human AD [16]. Interestingly, pharmacological inhibition of peroxisomal β-oxidation, resulting in VLCFA accumulation, is positively correlated with Aβ40 levels in rat cerebral cortex [66].

The relatively high CAT levels observed in 6-month-old Tg hippocampus are conceivably sustained by transcription factors activated in response to oxidative stress. To this respect, the cellular redox state sensor PGC-1α may play a role in regulating CAT expression [67]. Our immunohistochemical data support this relationship, in that at 6 months intense PGC-1α nuclear staining, suggestive of its induction and activity, is observed in CA1 pyramidal neurons, exclusively in Tg. The decrease in PGC-1α nuclear immunostaining seen at later AD stages, also parallels the behavior of CAT.

The same transcriptional co-activator, which has been involved in aging and age-associated diseases [68], may be responsible for the pattern of other antioxidant enzymes. Indeed, the increased PGC-1α nuclear immunoreactivity observed around 9–12 months in Wt neurons is paralleled by the raise in SOD1 and SOD2 levels, occurring in 12-month-old normal hippocampus. Conversely, the dramatically low expression of PGC-1α at 12 months in AD neurons, when we first observe hippocampal amyloid deposits (not shown), is concomitant with the lower levels of SOD1, SOD2 and, particularly, GPX1, compared to WT. Accordingly, altered glutathione redox status has been associated to the onset of amyloid plaques in a mouse model of AD [69] and decreased activities of SOD1 and GPX1 have been reported in symptomatic human AD [62,70]. By contrast, SOD2 overexpression in Tg mice results in diminished plaque formation [71].

In 18-month-old mouse hippocampus, genotype-dependent variations are observed for most peroxisomal and antioxidant proteins. At this stage, gliosis, known to occur during brain aging and exacerbated in AD [72-74], should be taken into account, when interpreting WB quantitative data. In fact, astrogliosis may be at least in part responsible for the peaks of PMP70 and Pex14p observed in old Tg mice. An important contribution to the overall brain peroxisomal population by astrocytes, highlighted by our GFAP/PMP70 double immunofluorescence images, is consistent with the notion that peroxisomes are abundant in this cell type [75-77]. Nevertheless, our immunohistochemical data on 18-month-old hippocampus clearly show enhanced positivity to PMP70 and Pex14p also in CA1 pyramidal neurons, markedly in the Tg, suggesting a global increase of peroxisome number in the hippocampal tissue, contributed by both neuronal and glial cells. Differently from the above membrane proteins, peroxisomal matrix enzymes, namely CAT, AOX and THL are not increased or even decreased, in 18-month-old hippocampus, possibly owing to age-related low efficiency of peroxisomal protein import [13] and in keeping with the impairment of brain peroxisomal acyl-CoA β-oxidation in physiological and pathological human aging [15,16].

Oxidative stress is known to occur in age-related neurodegeneration [78,79]. Our results on 18-month-old WT hippocampus show significantly lower levels of SOD1 and SOD2 proteins, while GPX1 is not decreased, with respect to previous ages. Thus, it seems that oxidative damage in the aging hippocampus may result from defective scavenging of superoxide anion, rather than hydrogen peroxide. In 18-month-old Tg hippocampus, GPX1 levels are significantly lower than in the corresponding WT, indicating impaired removal of H_2_O_2_, thus exacerbating the physiological age-related alteration of the cellular redox status. Concerning SODs, while SOD1 follows the decreasing trend of the age-matched WT hippocampus, SOD2 is not decreased in 18-month-old Tg, being significantly higher than in its WT counterpart. This finding can be explained by immunohistochemical data, showing particularly high concentration of SOD2 in glial cells surrounding amyloid plaques. Consistently, SOD2 - but not SOD1, CAT, or GPX1 - is induced in astroglia by activated microglia in an *in vitro* model of neuroinflammation [80].

A pro-oxidant cellular environment in the aged hippocampus is further demonstrated by the presence of oxidative damage markers (acrolein and 8-OH(d)G), particularly concentrated in Tg neurons. However, while in WT hippocampus both markers clearly show age-dependent increase, the same is not true for the Tg, where 8-OH(d)G immunoreactivity appears reduced in 18-month-old hippocampus, compared to the young. This observation is in agreement with data from other Authors indicating a negative correlation between 8-OH(d)G levels and histological amyloid burden [81,82].

## Conclusions

Overall, our results confirm and extend our previous observations on the early involvement of peroxisomes in AD pathogenesis [18]. In particular, we strongly suggest that these organelles constitute a first line of defense in support of mitochondria, primary targets of intracellular Aβ/ROS-mediated damage. This protective role specifically involves increased peroxisomal fatty acyl β-oxidation, likely providing mitochondria with acetyl-CoA and shortened acyl-CoAs. This conclusion may shed new light into the physiological significance of peroxisome metabolism in mature neurons, so far mainly related to the synthesis of plasma membrane components and to neurotransmission [7-10,83,84].

In brain aging, when oxidative stress *sensu strictu* begins, peroxisomes become target of ROS, their functions irreversibly decline, thus contributing themselves to the “pro-oxidant loop” [13]. Deepening knowledge of these processes, which are associated to aging and accelerated/exacerbated in AD, will add significantly to the dissection of pathogenic mechanisms of the disease. At the same time, the early response shown by peroxisomes supports the potential use of some of their proteins as biomarkers, to recognize the first signs of presymptomatic AD and to follow the effectiveness of novel therapeutic approaches, possibly involving peroxisomal induction. Indeed, preventing or treating the disease earlier than in its mild to moderate stages presently appears a major task, as stressed by the latest commentaries on the topic [85,86].

## Methods

### Animals

Heterozygous female Tg mice [20] and WT littermates were used for all experiments. Male mice (C57B6 × SJL), hemizygous for human AβPP695 carrying the double mutation K670N and M671L (FADSwedish mutation), were purchased from Taconic Farms, Inc. (Germantown, NY, USA). The Tg colony is maintained and the genotyping is performed as previously described [18].

For immunoblotting analyses, Tg and WT female mice aging 3, 6, 9, 12, and 18 months were used. Six animals from each group were killed by cervical dislocation, brains were rapidly excised on an ice-cold plate, and the hippocampus was dissected out before pooling samples (three pools of two hippocampi each). For morphological studies, three Tg mice and three WT littermates for each age considered were deeply anesthetized with urethane (1 g/kg body weight, injected i.p.), before rapid killing by transcardial perfusion with the fixative solution.

Experiments were performed in accordance with the European Community’s Council Directive 86/609/EEC. Formal approval of these experiments was obtained from the Italian Ministry of Health (D.L.vo 116/92; Prot. No. 155-VI-1.1.). All efforts were made to minimize the number of animals used and their suffering.

### Molecular analyses

#### Total homogenate preparation from hippocampal tissue

Protein extraction was performed by homogenizing tissue in lysis buffer (320 mM sucrose, 50 mM NaCl, 50 mM Tris–HCl, pH 7.5, 1% Triton X-100, 0.5 mM sodium orthovanadate, 5 mM β-glycerophosphate, 1% protease inhibitor), incubating on ice for 30 min. Homogenates were then centrifuged at 13,000 g for 10 min. The total protein content of the resulting supernatant was determined using a spectrophotometric assay, according to Bradford [87]. Samples were then diluted 3:4 in 200 mM Tris–HCl, pH 6.8, containing 40% glycerol, 20% β-mercaptoethanol, 4% sodium dodecyl phosphate (SDS), bromophenol blue.

#### Immunoblotting analyses

Western blotting (WB) experiments were performed as previously described [18]. Membranes were probed at 4°C overnight with the following primary antibodies: 1:10000 rabbit polyclonal anti-Pex14p (generous gift of Prof. R. Erdmann, Ruhr-Universität Bochum, Bochum, Germany); 1:1000 rabbit polyclonal anti-PMP70 (Sigma-Aldrich, St. Louis, MO, USA); 1:200 rabbit polyclonal anti-glial fibrillary acidic protein (anti-GFAP, Sigma-Aldrich); 1:1000 rabbit polyclonal anti-AOX (generous gift of Prof. A. Völkl, University of Heidelberg, Heidelberg, Germany); 1:1000 rabbit polyclonal anti-THL (generous gift of Prof. P. Van Veldhoven, Katholieke Universiteit Leuven, Leuven, Belgium); 1:2000 rabbit polyclonal anti-CAT (Rockland, Gilbertsville, PA, USA); 1:3000 rabbit polyclonal anti-GPX1 (Abnova, Taipei City, Taiwan); 1:1000 rabbit polyclonal anti-SOD1 (Abcam, Cambridge Science Park, Cambridge, UK); 1:10000 anti-SOD2 (Abcam); 1:3000 mouse monoclonal anti-β-actin (Sigma-Aldrich). This was followed by incubation with 1:2000 HRP-conjugated goat anti-rabbit or anti-mouse IgG secondary antibodies (Santa Cruz Biotechnology, Santa Cruz, CA, USA) in blocking solution, for 1 h at 4°C. Immunoreactive bands were visualized by a chemiluminescence detection kit (ECL Plus™ Western Blotting Detection Reagents, Amersham GE Healthcare, Little Chalfont, UK). The relative densities of immunoreactivity were determined by densitometry using the software ImageJ (NIH, Bethesda, MD, USA) and normalized with respect to β-actin. Data are mean of five different experiments.

### Morphological analyses

#### Brain tissue preparation

Mice were perfusion-fixed and brains were paraffin-embedded, as previously described [18]. The left halves from 3-month-old Tg and WT brains were used for immunoelectron microscopy.

#### Immunohistochemistry

Serial, 5-μm-thick sagittal brain sections from Tg and WT animals were deparaffinized and processed for immunohistochemistry, as previously described [18]. The following primary antibodies were used: 1:500 rabbit polyclonal anti-Pex14p (generous gift of Prof. R. Erdmann); 1:200 rabbit polyclonal anti-PMP70 (Sigma-Aldrich); 1:300 rabbit polyclonal anti-GFAP (Dako, Glostrup, Denmark); 1:100 rabbit polyclonal anti-AOX (generous gift of Prof. A. Volkl, Heidelberg); 1:200 rabbit polyclonal anti-CAT (Rockland); 1:100 rabbit polyclonal anti-SOD1 and 1:1000 anti-SOD2 (Abcam); 1:500 rabbit polyclonal anti-acrolein (generous gift of Prof. K. Uchida, Nagoya University, Nagoya, Japan); 1:200 rabbit polyclonal anti-PPARα (ABR Affinity BioReagents, Golden, CO, USA); 1:50 mouse monoclonal anti-PGC-1α (Calbiochem), and 1:500 mouse monoclonal anti-8OH(d)G (QED Bioscience Inc., San Diego, CA, USA),. In control sections, the primary antibody was omitted. Biotinylated goat anti-rabbit IgG (Vector Laboratories, Burlingame, CA, USA), or biotinylated goat anti-mouse IgG (Vector) were used as secondary antibodies. Immuno-complexes were visualized, and slides were dehydrated and mounted, as previously reported [18]. Sections were observed under an Olympus BX 51 microscope, equipped with a Leica DFC 420 camera; electronic images were captured by a Leica Application Suite system, and composed in an Adobe Photoshop CS5 format.

#### Immunofluorescence

For double immunofluorescence experiments, sagittal paraffin sections from 18-month-old Tg and WT brains were deparaffinized and rehydrated, as above. Sections were incubated overnight at 4°C with a mixture of rabbit polyclonal anti-PMP70 (1:100, Sigma-Aldrich) and either mouse monoclonal anti-GFAP (1:200, Chemicon, Temecula, CA, USA), or mouse monoclonal anti-Neuronal Nuclei (anti-NeuN 1:100, Chemicon). Sections were thoroughly rinsed with PBS, then incubated for 1 h at RT with a mixture of 1:100 Alexa488 conjugated goat anti-rabbit IgG (Invitrogen) and 1:500 Alexa555 conjugated goat anti-mouse IgG (Invitrogen). All antibodies were diluted with PBS containing 4% BSA. Triple immunofluorescence experiments were performed on 3-month-old Tg and WT brains, using a mixture of primary antibodies (1:100 rabbit polyclonal anti-PPARα, 1:200 sheep polyclonal anti-GFAP, 1:100 mouse monoclonal anti-NeuN) and of secondary antibodies (1:100 Alexa633 conjugated goat anti-rabbit IgG, 1:500 Alexa488 conjugated goat anti-mouse IgG, and 1:100 Alexa546 conjugated goat anti-sheep IgG). Controls were performed by omitting the primary antibody. Slides were observed in a Leica TCS SP5 confocal microscope and electronic images, captured by a Leica Application Suite system, were composed in an Adobe Photoshop CS5 format.

#### Immunoelectron microscopy

Samples from 3-month-old Tg and WT brains were sagittally cut by a vibratome, and 100-μm-thick sections, were processed for pre-embedding immunolocalization, according to the previously described procedure [18]. Sections were incubated with either 1:500 anti-PMP70 or with 1:150 anti-PPARα, as primary antibodies, diluted in PBS. After immunocomplexes visualization, slices were osmium-postfixed, dehydrated, and flat-embedded in Epon as previously described [18]. CA1 hippocampal fields of the resin-embedded sections, identified in a light microscope, were remounted on Epon blanks, and further sectioned on a Reichert Ultracut S ultramicrotome. Ultrathin sections were briefly contrasted with uranyl acetate and lead citrate, and observed in a Philips CM120 electron microscope equipped with a Philips Megaview III camera. Electronic images were captured by AnalySys 2.0 software and composed in Adobe Photoshop CS5 format.

### Statistical analysis

Statistical evaluation for WB experiments was performed using GraphPad Prism 4 software. For comparison between genotypes and among different ages, statistical analysis of WB densitometric values was accomplished using two-way analysis of variance (ANOVA) followed by the Bonferroni test to detect significant differences between groups. Means from independent experiments were then expressed as means ± SD. For all statistical analyses, P <0.05 was considered as statistically significant.

## Abbreviations

8-OH(d)G: 8-hydroxy(deoxy)guanosine;Aβ: Amyloid β;AD: Alzheimer’s disease;AOX: Acyl-CoA oxidase 1;CAT: Catalase;GFAP: Glial fibrillary acidic protein;GPX1: Selenium-dependent glutathione peroxidase;NeuN: Neuronal nuclei;Pex14p: Peroxin 14p;PGC-1α: Peroxisome proliferator-activated receptor γ coativator 1α;PMP70: Peroxisomal membrane protein of 70 kDa;PPAR: Peroxisome proliferator-activated receptor;PVDF: Polyvinylidene fluoride;RT: Room temperature;ROS: Reactive oxygen species;SDS: Sodium dodecyl phosphate;SOD1: CU,Zn-superoxide dismutase;SOD2: Mn-superoxide dismutase;Tg: Tg2576 transgenic mouse model of Alzheimer’s disease;THL: 3-ketoacyl-CoA thiolase;VLCFA: Very long chain fatty acid;WB: Western blotting;WT: Wild-type

## Competing interests

The authors declare that they have no competing interests.

## Authors’ contributions

FF made substantial contribution to conception and design of the study, carried out the morphological and molecular experiments and has been involved in drafting the manuscript. SS participated in performing immunoblotting experiments and statistical analyses. MD participated in interpretation of data and revised the manuscript critically. CB has been involved in morphological analysis and interpretation of data. LC participated in performing and interpreting immunofluorescence experiments. AC participated in the design of the study. FC revised the manuscript critically. MPC participated in design and coordination of the study and has been involved in revising the manuscript critically. SM conceived and designed the study, helped to draft the manuscript and gave final approval of the version to be published. All authors read and approved the final manuscript.
